# Selective Detection of Mg^2+^ for Sensing Applications in Drinking Water

**DOI:** 10.1002/chem.202201062

**Published:** 2022-07-08

**Authors:** Daniele Paderni, Eleonora Macedi, Larisa Lvova, Gianluca Ambrosi, Mauro Formica, Luca Giorgi, Roberto Paolesse, Vieri Fusi

**Affiliations:** ^1^ Department of Pure and Applied Sciences University of Urbino “Carlo Bo” Via della Stazione 4 I-61029 Urbino Italy; ^2^ Department of Chemical Sciences and Technology University of Rome “Tor Vergata” Via della Ricerca Scientifica 1 I-00133 Roma Italy

**Keywords:** chemosensors, fluorescence, magnesium, natural water analysis, user-friendly detection

## Abstract

A new series of ligands containing the 2‐(2‐hydroxy‐3‐ naphthyl)‐4‐methylbenzoxazole (HNBO) fluorophore showed selectivity for Mg^2+^ ions, without the interference of Ca^2+^. The most promising representative **L3** resulted the best performing sensor for Mg^2+^ both in solution and embedded in an all‐solid‐state optode, especially towards real samples of drinkable water.

## Graphical abstract

**PVC‐based solvent** polymeric membranes uploaded on cellulose‐based Color Catcher absorbent sheets were doped with a fluorescent chemosensor containing a polyamine unit decorated with the 2‐(2‐hydroxy‐3‐ naphthyl)‐4‐methylbenzoxazole (HNBO) fluorophore to develop user‐friendly disposable all‐solid‐state devices for the selective detection of Mg^2+^ in real samples of commercial and tap drinking water, without the interference of Ca^2+^and other Alkali (A) and Alkaline‐Earth (AE) metal ions.

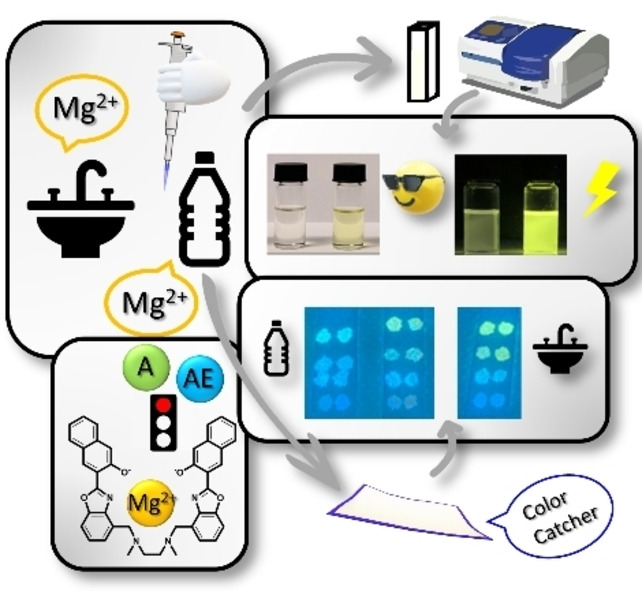

The selective detection of Mg^2+^ in biological and environmental samples arouses great interest in many scientific fields, due to both the crucial role it plays in all living beings as well as the fact that too high or too low levels of Mg^2+^ could be harmful to animals and humans.[^1^, ^2^, ^3^]

Among the analytical procedures able to track Mg^2+^ ions, the employment of optical chemosensors represents an efficient strategy that offers advantages on instrumental methods and rests on simplicity, reliability, velocity and possibility to operate in real time conditions.[^4^, ^5^, ^6^, ^7^, ^8^, ^9^, ^10^] However, most chemosensors reported so far are not able to distinguish between Mg^2+^ and other metal cations, especially Ca^2+^ and Zn^2+^.[^11^, ^12^, ^13^, ^14^, ^15^, ^16^, ^17^, ^18^] The availability of chemosensors that offer selectivity for Mg^2+^ vs. Ca^2+^ thus represents a remarkable analysis tool, both for biological and environmental samples. To this purpose, the development of all‐solid‐state, disposable and low‐cost optical sensors permitting the fast and selective Mg^2+^ detection without the employment of expensive equipment, complex sample preparations and skilled operators involvement is an attractive, as far a challenging analytical task.

Pursuing the aim to develop selective optical chemosensors for Mg^2+^, a new series of ligands containing the 2‐(2‐hydroxy‐3‐naphthyl)‐4‐methylbenzoxazole (HNBO)^[19]^ fluorophore linked to different aliphatic amine chains was synthesized (Figure 1).

**Figure 1:**
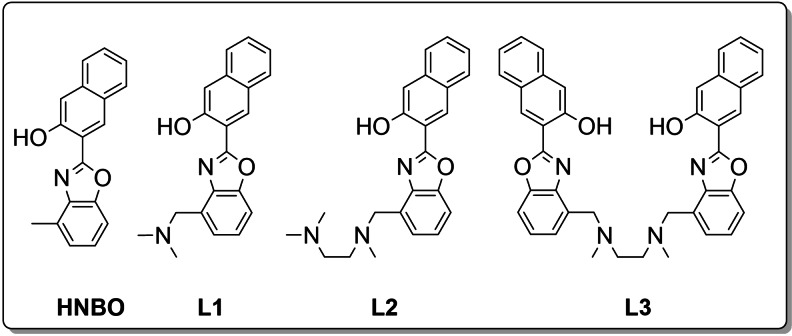
HNBO and HNBO‐based ligands **L1‐L3** synthesized in this study.

The series was designed with a growing degree of structural complexity, moving from the simple HNBO to ligands containing one HNBO unit linked to a dimethylamine (N‐(2‐(2’‐hydroxy‐3’‐naphthyl)benzoxazol‐4‐ylmethyl)‐N,N‐dimethylamine, **L1**) or a N,N,N’‐Trimethylethylenediamine fragment (N‐(2‐(2’‐hydroxy‐3’‐naphthyl)benzoxazol‐4‐ylmethyl)‐N,N’,N’‐trimethylethylendiamine dihydrochloride, **L2 ⋅ 2HCl**) or two HNBO units linked to a N,N’‐Dimethylethylenediamine fragment (N,N’‐bis(2‐(2’‐hydroxy‐3’‐naphthyl)benzoxazol‐4‐ylmethyl)‐N,N’‐dimethylethylendiamine, **L3**).

Briefly, ligands **L1**‐**L3** were synthesized via a nucleophilic substitution between the amine fragment and the fluorophore, prior bromination at the benzylic position of HNBO (for more details see the Supporting Information, Scheme S1). The latter was previously synthesized as reported in the literature.^[20]^

HNBO and the three ligands were mainly tested towards Alkali and Alkaline‐earth ions (A and AE in the following; Li^+^, Na^+^, K^+^, Cs^+^, Mg^2+^, Ca^2+^, Sr^2+^, Ba^2+^) in DMSO+1.5 % H_2_O solution, at *I=*1.2 ⋅ 10^−3^ mol dm^‐3^ NMe_4_Cl, and containing an equimolar amount of tetramethylammonium hydroxide (TMAOH) by spectrophotometric and spectrofluorimetric measurements. Among the tested A and AE cations **L1**‐**L3** only responded to Mg^2+^, while the sole HNBO fluorophore did not respond to any of them (Figures 2, S1–S4).

**Figure 2:**
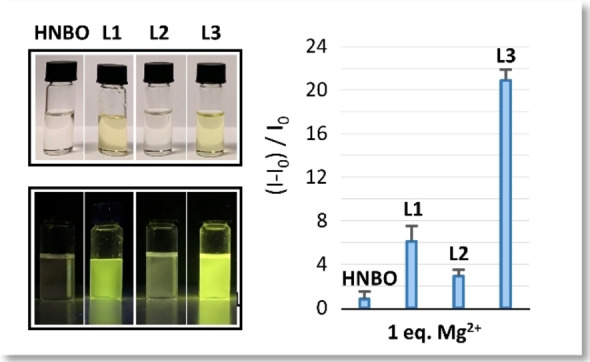
Maximum emission intensity of HNBO and **L1**‐**L3** upon addition of 1 equiv. of Mg^2+^ (λ_ex_ = 440 nm, λ_em_=530 nm (HNBO), 540 nm (**L1**), 535 nm (**L2**), 537 nm (**L3**)) and colour change (top) and enhancement of the fluorescence under a 365 nm UV lamp (bottom) after the addition of 1 equiv. of Mg^2+^ to HNBO and **L1**‐**L3**. [**L**]=1.2 ⋅ 10^−5^ mol dm^−3^ DMSO+1.5 % H_2_O; *I=*1.2 ⋅ 10^−3^ mol dm^−3^ NMe_4_Cl. Number of replicas: 3.

Interestingly, among the three ligands **L3** showed the highest emission increase in the presence of Mg^2+^, attributed to the coordination of the cation (chelation enhancement of the fluorescence, CHEF effect, Figure 2), considering all A and AE and, noteworthy, some transition metal ions that could possibly interfere in the detection of Mg^2+^ in aqueous medium (Zn^2+^, Cd^2+^ and Pb^2+^; Figures 4a and S5). For this reason, in this contribution the studies performed on **L3** are going to be described more in depth.

The UV‐Vis absorption titration with Mg^2+^ showed the growth of a new absorption band at 440 nm, ascribable to the deprotonation of the naphthol moiety favored by the coordination of the cation (Figure 3a). The absorption increase parallels the great enhancement of the emission intensity at 537 nm that occurs upon addition of Mg^2+^ (Φ=0.09 (free ligand), 0.12 (upon addition of 1 equiv. Mg^2+^), λ_ex_=440 nm, Figure 3b). Both measurements revealed the formation of a species with a 1 : 1 ligand to Mg^2+^ molar ratio.

**Figure 3:**
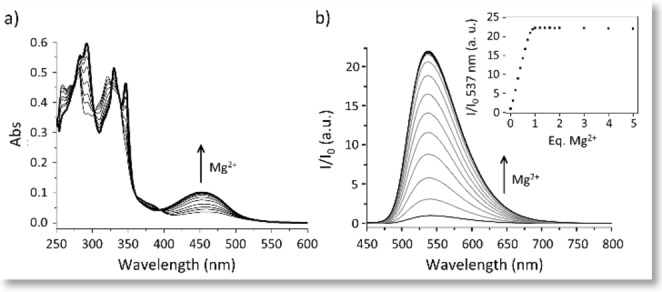
UV‐Vis absorption (a) and emission (b) spectra of **L3** (1.2 ⋅ 10^−5^ mol dm^−3^) in DMSO+1.5 % H_2_O, *I=* 1.2 ⋅ 10^−3^ mol dm^−3^ NMe_4_Cl upon addition of up to 5 equiv. Mg^2+^. λ_ex_=440 nm.

^1^H NMR titration with Mg^2+^ confirmed the formation of a mononuclear complex, indeed the spectrum did not show any variation following the addition of 1 equiv. of Mg^2+^ (Figure S7). More in detail, moving from the (H _2_ **L3**)^2−^ species to the 1 : 1 complex, all aromatic resonances shift downfield, whereas in the aliphatic region the resonance of H12 shifts upfield and those of H11 and H13 split in characteristic AB systems, suggesting the stiffening of the structure upon the ion complexation (Figure S8). Moreover, since a *C*_2_ symmetry on the NMR time scale is observed, a cooperation between the two fluorophore moieties in the Mg^2+^ complexation can be suggested.

**L3** is a possible ESIPT‐based sensor:^[21]^ if this was the case, the metal coordination would suppress the ESIPT mechanism, resulting in an hypsochromically shifted *enol*‐fluorescence, and ratiometric signals could be achieved. Since no ratiometric response was observed in this case, another mechanism is to be taken into account. Considering all data and the behavior of **L3** at different pH fields (Figure S6), TICT seems to be the prevailing quenching mechanism from acid to neutral pH field, more than PET, while, at basic pH, the deprotonation of HNBO increases the conjugation of the π‐system preventing the TICT and PET processes, switching ON the emission. Similarly, the coordination of Mg^2+^ at neutral pH favors the rings conjugation, preventing the TICT and the possible PET quenching[^19^, ^20^] affording a highly emitting species (see Supporting Information for more details).

A remarkable fluorescence selectivity of **L3** for Mg^2+^ vs. all tested metal ions was observed: among the tested A and AE, only Mg^2+^ caused indeed a pronounced CHEF effect (Figure 4a, blue bars).

**Figure 4:**
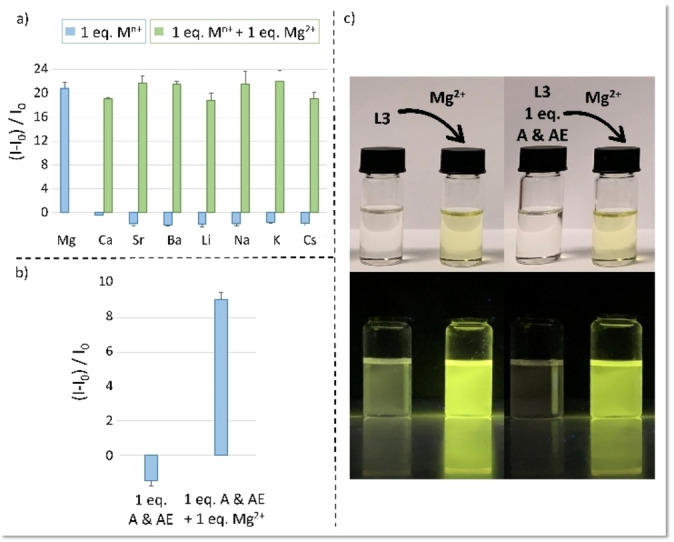
Maximum emission intensity of **L3** upon addition of a) 1 equiv. of A and AE or b) an equimolar mixture of A & AE, and following further addition of 1 equiv. of Mg^2+^ ( λ_ex_=440 nm; λ_em_=537 nm). c) Colour change (top) and enhancement of the fluorescence under a 365 nm UV lamp (bottom) after the addition of 1 equiv. of Mg^2+^ to **L3** or an equimolar **L3**/A & AE mixture. [**L3**]=1.2 ⋅ 10^−5^ mol dm^−3^ DMSO+1.5 % H_2_O; *I=*1.2 ⋅ 10^−3^ mol dm^−3^ NMe_4_Cl. Number of replicas: 3.

The presence in solution of any A and AE did not hamper the fluorescence response of **L3** to Mg^2+^, neither individually (Figure 4a, green bars) nor pooled in a solution simulating drinkable water (Figure 4b), indicative of a non‐competitive behavior. ^1^H NMR measurements revealed an interaction between **L3** and Ca^2+^ (generally a strong Mg^2+^ competitor) (Figure S8), but no variation in the emission behavior of the system was observed, as above reported (Figure 4a, blue bars). The addition of Mg^2+^ to the Ca^2+^‐**L3** solution switched‐ON the emission and produced the ^1^H NMR spectrum of the Mg^2+^‐complex (Figure S8), highlighting the better affinity of Mg^2+^ for the chemosensor and the higher stability of the Mg^2+^‐complex compared to the others (Figure 4a, green bars).

The formation of the Mg^2+^‐complex in both the absence and presence of an equimolar A and AE mixture is visible to the naked eye via both a color change of the solution from colorless to yellow as well as a fluorescence increase under a common 365 nm UV lamp (Figure 4c).

The ability of **L3** to respond to Mg^2+^ ions in real samples was assessed by analyzing different commercial and tap water samples. To this aim, little amounts of the water samples were added to a DMSO solution of **L3**, along with distilled water as a comparison. The system proved to work well with the real samples, responding consistently with the Mg^2+^ content of commercial and tap waters (Figure 5), regardless of the complex mixture of cations (including Ca^2+^) and anions present in solution. Also in this case, the formation of the Mg^2+^‐complex is visible both via colorimetric and fluorimetric change (Figure 5).

**Figure 5:**
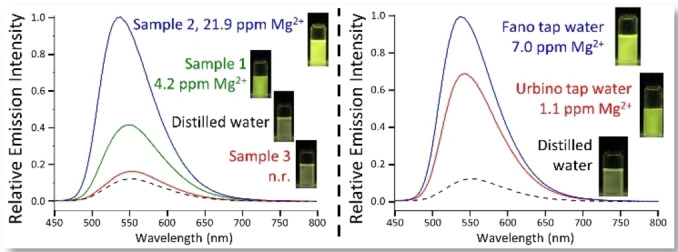
Real samples analysis on commercial drinking water (left) and tap water samples (right) by using **L3**. 37.5 μL of water samples (1.5 %) were added to a DMSO solution of **L3**. [**L3**]**=**1.2 ⋅ 10^−5^ mol dm^‐3^ DMSO, *I=*1.2 ⋅ 10^−3^ mol dm^−3^ NMe_4_Cl. The Mg^2+^ content of each sample is reported, along with the fluorescence emission under a 365 nm UV lamp.

The normalized intensity emission at 537 nm of samples doped with Mg^2+^ increased with the Mg^2+^ concentration up to 20 ppm then the system plateaued. The statistical analysis of the trend through the linear regression method^[22]^ furnished a limit of detection (LOD) of 1.0 ppm (6.0 ⋅ 10^−7^ mol dm^−3^), a limit of quantification (LOQ) of 3.5 ppm (2.1 ⋅ 10^−6^ mol dm^−3^) and a limit of linearity (LOL) in the 0–20 ppm range (1.2 ⋅ 10^−5^ mol dm^−3^) of magnesium (R=0.99) (Figure S9).

In light of the promising sensing behavior of **L1‐L3** in solution, the possibility to develop all‐solid‐state optodes for a fast and inexpensive Mg^2+^ detection was investigated by employing PVC‐based solvent polymeric membranes doped with **L1‐L3** uploaded on two different solid supports: Whatman 1400 filter paper (FP) and commercially available cellulose‐based Color Catcher absorbent sheets (CC). Membranes of total 100 mg weight were prepared according to a common procedure^[19]^ (Mb**L**.1 and Mb**L**.2 (**L**=**L1‐L3**); see the Supporting Information for more details) and their compositions are listed in Table S1.

The membranes were doped with a lipophilic cation‐exchanger (potassium tetra‐p‐chlorophenyl borate, TpClPBK) to promote the analyte ions flux into the membrane, favoring the deprotonation of the naphtholic ‐OH groups of **L1‐L3** and the coordination of A and AE hard metals. Moreover, the small amount of lipophilic anionic TpClPB^−^ sites stabilizes the membrane properties through keeping the overall electroneutrality. The ligand/cation‐exchanger ratios were selected based on the formation of 1 : 1 ligand/Mg^2+^ complexes; variable amounts of TpClPBK with respect to each ligand were tested (Table S1), with the highest quantity of exchanger chosen in accordance with the number of acidic groups in **L1‐L3** (1, 3 and 2 equiv. for **L1**, **L2 ⋅ 2HCl** and **L3**, respectively).

Arrays of sensing spots of 7 columns x 6 (A) or 5 (AE) lines size deposited on FP or CC support were prepared to test the optodes response towards A, AE and some transition metal ions (Zn^2+^, Cd^2+^ and Pb^2+^; metal ions added as chloride or nitrate salts) by direct application of a drop of an aqueous solution of the analyte over the sensing spots in a concentration range from 1 ⋅ 10^−6^ to 1 ⋅ 10^−1^ mol dm^−3^ (Figures 6, S10, S11).

**Figure 6:**
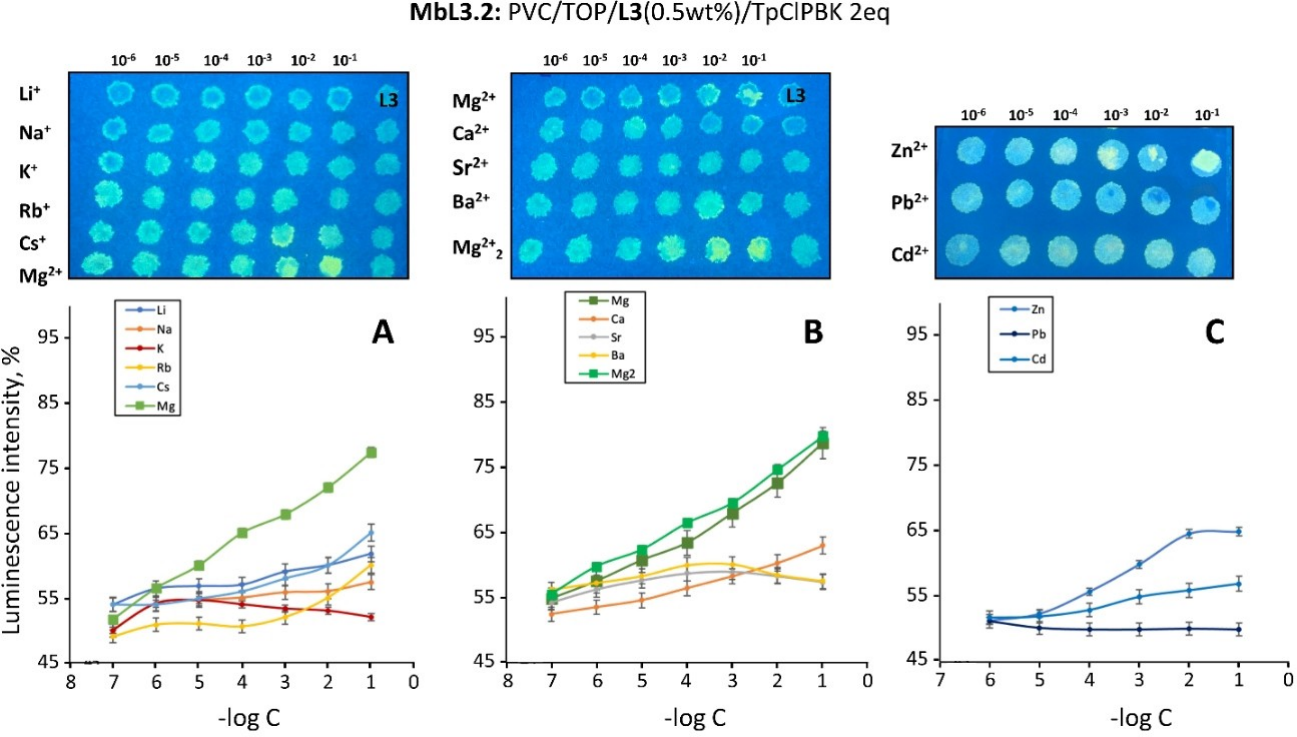
The response of **L3**‐based optical sensing spot arrays to a) Alkali, b) Alkaline‐Earth and c) Zn^2+^, Pb^2+^ and Cd^2+^ metal cations, (λ_ex_=365 nm). Top: photographs of sensing spots deposited on CC support; bottom: calibration curves representing the relative luminescence intensity (in %) of Mb**L3**.2‐based optode to growing concentrations (‐log C) of tested ions. Number of replicas: n=6.

For an optical response quantification, pictures of the optodes illuminated at 365 nm were taken with a smartphone at 10 cm distance, then the color variations were digitalized with in‐house‐written Matlab codes (v. 7.9, 2009, codes. The MathWorks, Inc., Natick, USA). The optodes response upon the analyte addition was converted into three main colors of the visible spectrum (red (630 nm), green (530 nm) and blue (480 nm)), according to the RGB scale, and the luminescence intensity of each sensing spot was calculated according to Equation 1:

(1) $$I=\left(R+G+B\right)/\left(3{}^{*}255\right)$$

where R, G and B represent the luminescence intensities at RGB channels, while value 255 is the maximum intensity of the optical signal measured with the smartphone detector. The RGB values were extracted at the center of every single sensing spot in at least 3 replicas and evaluated after subtraction of the intensities of the spot without both the analyte and the FP or CC support background.

In accordance with the above described studies in solution, the **L1‐L3**‐based membranes showed a pronounced selectivity for Mg^2+^ over all other tested metal ions, displaying a significative naked‐eye visible increase in luminescence starting from [Mg^2+^]=1 ⋅ 10^−3^ mol dm^−3^ (Figures 6, S10, S11). Among the tested membranes, the preliminary tests have shown the highest response toward Mg^2+^ for PVC‐based membranes deposited on CC solid support and for those featuring a stoichiometric amount of cation exchanger compared to the acidic functions of the ligand (Mb**L**.2, **L**=**L1‐L3**) (Figures 7 and S12). More in particular, Mb**L3**.2, even if resulted partially luminescent itself, showed the widest linear range of luminescence response to Mg^2+^ registered as luminescence optical intensity, I, calculated as from Equation (1) (Figure 6a,b).

**Figure 7:**
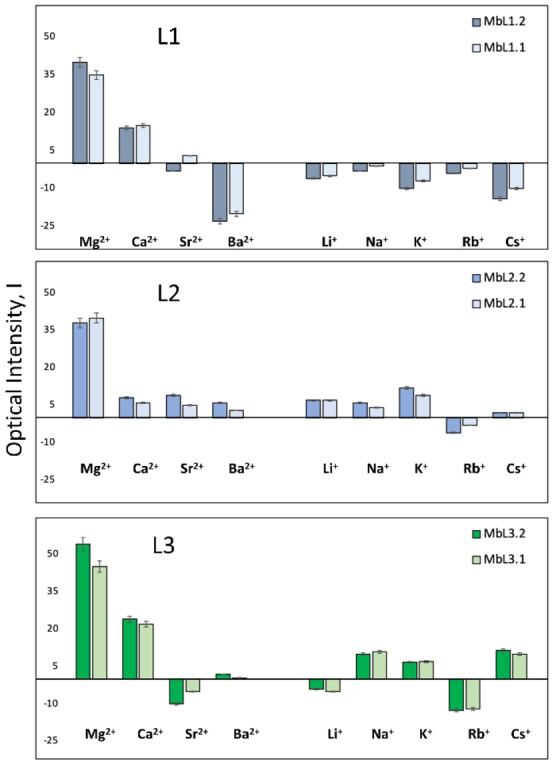
Optical response of **L1‐L3**‐based membranes in individual 0.01 mol dm^−3^ aqueous solutions containing A and AE. Luminescence evaluated at 365 nm.

No influence of other A and AE were registered, except for a partial response of **L1**‐ and **L3**‐doped membranes to high concentrations of Ca^2+^ (Figure 7).

Zn^2+^, Cd^2+^ and Pb^2+^ were also tested, revealing a pronounced response of **L1**‐ and **L2**‐based membranes to high concentrations of Zn^2+^ and Cd^2+^, whereas only a modest response of **L3**‐based optodes to Zn^2+^ was registered (Figures 6c, S10c, S11c). Competition tests between Cd^2+^/Mg^2+^ or Zn^2+^/Mg^2+^ ions showed almost no influence of these interfering ions (1 ⋅ 10^−5^ mol dm^−3^) on the **L3**‐based optodes response toward Mg^2+^ (concentration range 1 ⋅ 10^−5^–1 ⋅ 10^−1^ mol dm^−3^ for Cd^2+^, 1 ⋅ 10^−4^–1 ⋅ 10^−1^ mol dm^−3^ for Zn^2+^, Figure S13).

At higher concentration (1 ⋅ 10^−3^ mol dm^−3^), the interfering Cd^2+^ and Zn^2+^ ions showed more influence on the **L3**‐based optodes response to target Mg^2+^ ions. Nevertheless, taking into account that the amount of Cd^2+^ and Zn^2+^ ions in drinking waters is commonly low and must be well controlled according to the WHO guideline for potable water,^[23]^ they should not interfere with the Mg^2+^ assessment by the developed optodes.

Moreover, since the pH of drinking water may vary in a quite wide range (from 5 to 8.5 pH units), the pH influence on **L3**‐based optodes response toward Mg^2+^ ions has been investigated on different backgrounds (0.01 mol dm^‐3^ MES, HEPES and PBS buffer solutions with pH 5.5, 7.5 and 8.6 respectively, and tap water with pH 8.1). The results revealed no significant pH influence on **L3**‐based optodes luminescence response to Mg^2+^ ions in the entire tested concentration range (1 ⋅ 10^−6^–1 ⋅ 10^−1^ mol dm^−3^; Figure S14a) and for all the calibration curves the linear trend and the slope remain indeed the same (Figure S14b).

The disposable fluorescent sensors were hence employed for the detection of the Mg^2+^ content in solutions simulating natural waters and containing all A and AE in various concentrations (10^−5^, 10^−4^, 10^−3^ mol dm^−3^) as far as in mineral waters. Disposable CC strips (approximately 0.9 x 3 cm size) with deposited a small sensor array formed by four sensing membranes (Mb**L1**.2, Mb**L2**.2, Mb**L3**.1 and Mb**L3**.2) replicated in two spots, were employed in these analyses (Figure S15a). A clear difference in the optical luminescence response of the sensor array in multicomponent model solutions in the presence of Mg^2+^ was observed (Figure S15a). Moreover, the application of a PCA analysis to the numerical outputs of sensor array luminescence response in terms of RGB intensities permitted to clearly identify all multi‐component model solutions in the presence and absence of Mg^2+^ ions (1 ⋅ 10^−2^ mol dm^−3^, Figure S15b).

Finally, the performance of the most promising Mb**L3**.2 membrane, preliminarily calibrated in individual solutions of Mg^2+^ (10^−6^–10^−1^ mol dm^−3^ range), was tested for the Mg^2+^ assessment in tap, distilled and mineral waters featuring high and low magnesium content (Figure 8). Results in mineral waters were in a very good agreement with data provided by the producers, with a mean relative error (R%) lower than 5 %, indicating the efficacy of the developed optical platform.

**Figure 8:**
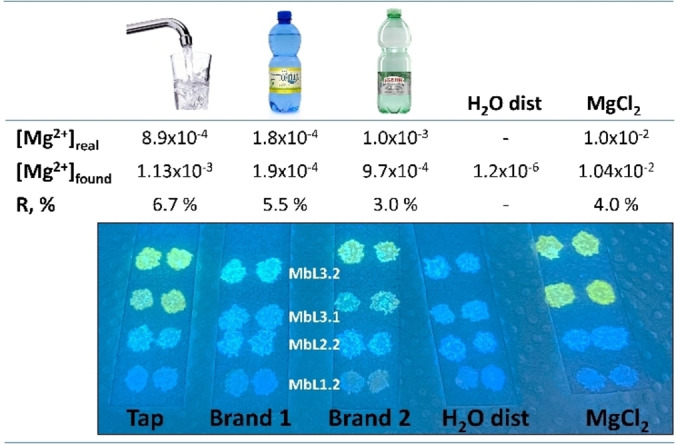
Real water samples analysis by using all‐solid‐state optodes. On the top are listed the [Mg^2+^]_real_ provided by the producer (or available on ACEA site for tap water^[24]^), the [Mg^2+^]_found_ determined by Mb**L3**.2 and the relative error, R%. Photograph taken by a smartphone upon illumination at λ_ex_=365 nm (standard laboratory UV‐lamp).

In conclusion, among three new HNBO‐based ligands, **L3** proved to bind Mg^2+^ in solution in a 1 : 1 molar ratio with a highly selective fluorescence response to Mg^2+^ also in the presence of Ca^2+^ and other Alkali and Alkaline‐earth metal ions.

**L3** also proved as the most promising candidate for the development of all‐solid‐state optodes as advantageous robust, affordable, sensitive, specific, user‐friendly and equipment‐free devices for magnesium detection. Both in solution and in the solid optical platform, **L3** works as a probe for metal ion‐induced chromo‐/fluorogenic dual signaling of Mg^2+^ both in artificial and real water samples for human consumption.

## Conflict of interest

There are no conflicts to declare.

## Supporting information

As a service to our authors and readers, this journal provides supporting information supplied by the authors. Such materials are peer reviewed and may be re‐organized for online delivery, but are not copy‐edited or typeset. Technical support issues arising from supporting information (other than missing files) should be addressed to the authors.

Supporting Information

## Data Availability

The data that support the findings of this study are available from the corresponding author upon reasonable request.
