# Supplementary material for: Selective Detection of Mg2+ for Sensing Applications in Drinking Water
Source: Chemistry. 2022 Jul 8;28(49):e202201062. doi: 10.1002/chem.202201062 (PMC9542287; doi:10.1002/chem.202201062)
Supplement: Supplementary file 1 — Supporting Information [file CHEM-28-0-s001.pdf]

Chemistry–A European Journal

Supporting Information

Selective Detection of Mg^{2+} for Sensing Applications in Drinking Water

Daniele Paderni, Eleonora Macedi,* Larisa Lvova,* Gianluca Ambrosi, Mauro Formica, Luca Giorgi, Roberto Paolesse, and Vieri Fusi*

Content

Instruments and materials 2
Synthesis and Characterization of ligands L1-L3 3
UV-Vis and fluorescence emission measurements 5
NMR spectroscopy 10
Determination of LOD, LOQ and LOL 12
Membranes preparation 12
Chemometric data treatment 14

Instruments and materials

All chemicals were purchased in the highest quality commercially available. The solvents were RP grade, unless otherwise indicated, and used without further purification. All reactions involving moisture-sensitive reagents were carried out under a nitrogen atmosphere using standard vacuum line techniques and glassware that was flame-dried before use.
Elemental analyses were performed with a Thermo Finnigan Flash 1112 EA CHN analyser.
Mass spectra were performed with an Agilent 1200 Series HPLC system equipped with a binary pump and a C18 column (Phenomenex Synergi™ 4 μm Fusion-RP 80 Å, LC Column 50 x 2 mm, Ea), coupled to a SCIEX mod. API 4000 QqQ triple quadrupole mass spectrometer with ESI source.
Ion Chromatography was performed by using a Metrohm Eco IC equipped with a Metrosep C4 column for cations.
Pictures depicted in Fig. 2, 4, 5, 6, S10, S11, S13, S14 and S15 were taken with the digital camera of a common smartphone.

Synthesis and Characterization of ligands L1-L3

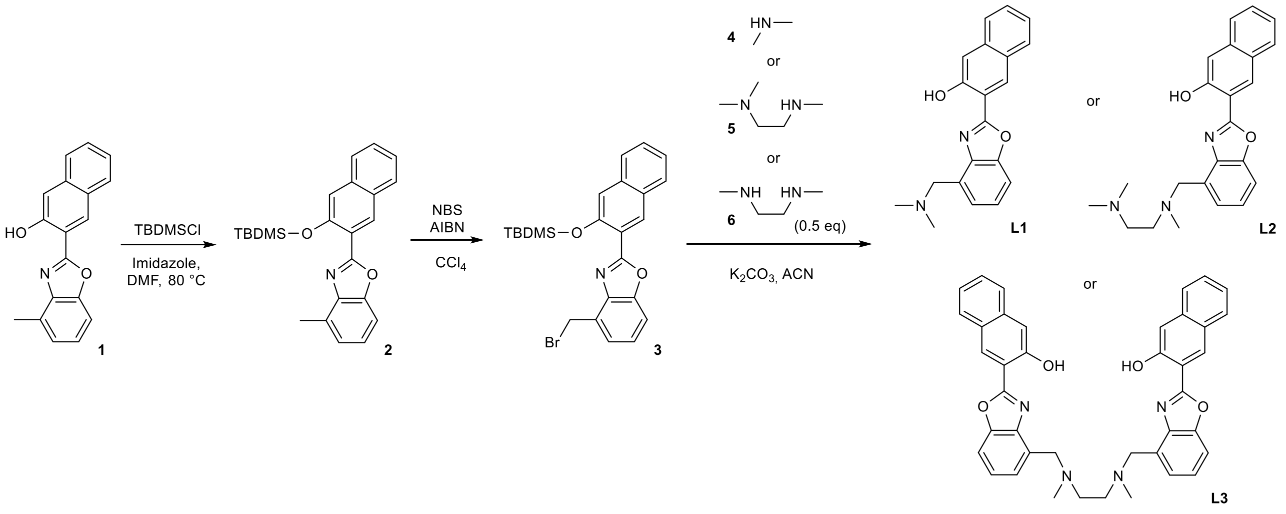

Scheme S1. Synthetic procedure to obtain ligands **L1-L3.**

Compound **1** (HNBO) was synthesized according to a previously reported procedure (see ref. 20, main text).

(2-(2'-tert-Butyldimethylsilyl)oxy-3'-naphtyl)-4-methylbenzoxazole (2)

A solution of imidazole (1.24 g, 0.0182 mol) in N,N-dimethylformamide (20 cm^3) was added dropwise in an inert atmosphere to a solution containing **1** (2.50 g, 9.08 mmol) and tert-butyldimethylchlorosilane (TBDMSCl, 2.05 g, 0.0129 mol) in N,N-dimethylformamide (130 cm^3). The mixture obtained was stirred at 80 °C for 24 h and then added of a 5% hydrogen carbonate aqueous solution (150 cm^3). The obtained mixture was extracted with dichloromethane (6 × 50 cm^3) and washed with brine (3 × 50 cm^3); the extracted organic phases were combined, dried on sodium sulfate and evaporated under vacuum to obtain **2** as a crystalline solid (2.90 g, 82%). ^{1}H NMR (400 MHz, $CDCl_3$, 25°C) δ = 0.27 (s, 6H), 1.06 (s, 9H), 2.72 (s, 3H), 7.17-7.33 (m, 3H), 7.39-7.43 (m, 2H), 7.50-7.53 (t, 1H), 7.71-7.73 (d, 1H), 7.89-7.92 (d, 1H), 8.65 (s, 1H) ppm. Anal. for $C_{24}H_{27}NO_2Si$ (389.57): Calcd C 74.00, H 6.99, N 3.60; Found C 73.8, H 7.1, N 3.7.

4-Bromomethyl-2-(2'-tert-Butyldimethylsilyl)oxy-3'-naphthyl)-benzoxazole (3).

α,α'-Azoisobutyrronitrile (AIBN, 0.11 g, 0.70 mmol) was added under an inert atmosphere to a refluxing and stirred solution of **2** (2.09 g, 5.36 mmol) and N-bromosuccinimide (NBS, 1.31 g, 6.97 mmol) in CCl_4 (100 cm^3). The resulting mixture was stirred with reflux overnight, then cooled to room temperature and filtered. The filtrate was concentrated under vacuum and subsequently added of n-hexane and placed in the refrigerator, obtaining a precipitate. The white solid of **3** was collected by filtration (1.35 g, y = 54%). ^{1}H NMR (400 MHz, $CDCl_3$, 25°C) δ = 0.31 (s, 6H), 1.09 (s, 9H), 5.00 (s, 2H), 7.18-7.73 (m, 6H), 7.32-7.53 (m, 6H), 7.72 (d, 1H), 7.90

(d, 1H), 8.71 (s, 1H) ppm. Anal. for $C_{24}H_{26}BrNO_2Si$ (468.47): Calcd C 61.53, H 5.59, N 2.99; Found C 61.3, H 5.7, N 3.1.

N-(2-(2'-hydroxy-3'-naphthyl)benzoxazol-4-ylmethyl)-N,N-dimethylamine (L1), N-(2-(2'-hydroxy-3'-naphthyl)benzoxazol-4-ylmethyl)-N,N',N'-trimethylethylendiamine dihydrochloride (L2·2HCl), N,N'-bis(2-(2'-hydroxy-3'-naphthyl)benzoxazol-4-ylmethyl)-N,N'-dimethylethylendiamine (L3).

A solution of **3** (0.40 g, 1.0 mmol) dissolved in 20 ml of acetonitrile was added dropwise under nitrogen to an acetonitrile solution (20 cm^3) containing K_2CO_3 (0.41 g, 3.0 mmol) and 40% aqueous dimethylamine (**4**, 0.12 cm^3, 1.0 mmol) for **L1** or N,N,N'-trimethylethylenediamine (**5**, 0.13 cm^3, 1.0 mmol) for **L2** or N,N'-dimethyl ethylenediamine (**6**, 0.053 cm^3, 0.5 mmol) for **L3**. The reaction mixture was stirred at room temperature overnight. The remaining white precipitate was filtered off and the filtrate was concentrated under vacuum, obtaining a yellow sticky oil.

L1: The obtained oil was triturated with n-hexane at room temperature. The yellow solid was collected by filtration (0.19 g, y = 61%). ^{1}H NMR (400 MHz, d_6-DMSO, 25°C) δ = 2.25 (s, 6H), 3.84 (s, 2H), 6.99 (s, 1H), 7.04 (t, J = 7.3 Hz, 1H), 7.28 (t, J= 7.6 Hz, 1H), 7.38 (m, 2H), 7.47 (d, J = 8.1 Hz, 1H), 7.65 (m, 1H), 7.77 (d, J = 8.1 Hz, 1H), 8.40 (s, 1H) ppm. ^{13}C NMR (100 MHz, d_6-DMSO, 25°C) δ = 45.7, 58.2, 109.5, 111.7, 120.9, 125.0, 125.2, 127.7, 129.1, 130.6 ppm. MS (ESI): m/z = 318.14 $[M{-}H]^+$. Anal. for $C_{20}H_{18}N_2O_2$ (318.4): Calcd C 75.45, H 5.70, N 8.80; Found C 75.3, H 5.8, N 8.9.

L2·2HCl: The pure **L2** was obtained as hydrochloride salt by the addition of a 10% ethanolic solution of HCl to an ethanolic solution (1 cm^3) of the crude obtaining 0.24 g of yellow solid (y = 54%). ^{1}H NMR (400 MHz, d_6-DMSO, 25°C) δ = 2.14 (s, 6H), 2.24 (s, 3H,), 3.92 (s, 2H), 6.39 (s, 1H), 6.65 (td, J_1 = 7.4, J_2 = 1.1 Hz, 1H), 6.99 (td, J_1 = 7.5, J_2 = 1.3 Hz, 1H), 7.11 (d, J = 8.4 Hz, 1H), 7.26 (t, J = 7.6 Hz, 1H), 7.32 (dd, J_1 = 7.7, J_2 = 1.2 Hz, 1H), 7.43 (d, J = 8.2 Hz, 1H), 7.51 (dd, J_1=7.7, J_2 = 1.3 Hz, 1H), 8.06 (s, 1H) ppm. ^{13}C NMR (100 MHz, d_6-DMSO, 25°C) δ = 131.4, 128.8, 126.1, 124.0, 123.7, 123.4, 116.9, 112.1, 108.9, 57.7, 56.6, 55,4, 42.9, 46.0 ppm. MS (ESI): m/z = 376.2 $[M{-}H]^+$. Anal. for $C_{23}H_{27}Cl_2N_3O_2$ (448.4): Calcd C 61.61, H 6.07, N 9.37; Found C 61.4, H 6.2, N 9.5.

L3: The obtained oil was triturated with n-hexane at room temperature. The yellow solid was collected by filtration (0.16 g of **L3** as a yellow solid (y = 50%). ^{1}H NMR (400 MHz, d_6-DMSO, 25°C) δ = 2.25 (s, 6H), 2.69 (s, 4H), 3.96 (s, 4H), 6.35 (s, 2H), 6.61 (td, J_1 = 7.3, J_2 = 1.1 Hz, 2H), 6.97 (td J_1 = 7.6, J_2 = 1.3 Hz, 2H), 7.10 (d, J=8.1 Hz, 2H), 7.25 (t, J = 7.8 Hz, 2H), 7.34 (dd, J = 7.3 Hz, 2H), 7.41 (d, J = 7.8 Hz, 2H), 7.50 (d, J = 7.8 Hz, 2H), 8.06 (s, 2H) ppm. ^{13}C NMR (100 MHz, d_6-DMSO, 25°C) δ = 43.0, 55.7, 56.8, 108.8, 112.1, 116.6, 123.5, 123.7, 124.0, 126.0, 128.9, 131.4 ppm. MS (ESI): m/z = 635.26 $[M{-}H]^+$. Anal. for $C_{40}H_{34}N_4O_4$ (634.7): Calcd C 75.69, H 5.40, N 8.83; Found C 75.5, H 5.5, N 8.9.

UV-Vis and fluorescence emission measurements

The spectrophotometric measurements were carried out at 298.1 K using a Varian Cary-100 spectrophotometer equipped with a temperature control unit**.** Uncorrected emission spectra were obtained with a Varian Cary Eclipse fluorescence spectrophotometer. Luminescence quantum yields were determined using 2,2'-biphenol in acetonitrile (Φ = 0.29).[1]

All UV–Vis and fluorescence emission measurements were performed in DMSO + 1.5% H_2O (I= $1.2\cdot10^{-3}$ mol dm^{-3} NMe_4Cl) solutions containing an equimolar amount of tetramethylammonium hydroxide (TMAOH: 1 eq. for **L1**; 3 eq. for **L2·2HCl**; 2 eq. for **L3**) at 25 °C, with a ligand concentration of $1.2\cdot10^{-5}$ mol dm^{-3} and by exciting at λ_{ex} = 440 nm. UV-Vis and fluorescence emission spectra of **L1**, **L2** and **L3** are depicted in Figure S1-S3 and Figure 3 (main text).

Screenings with Alkali and Alkaline-earth metal ions (A and AE) were carried out by adding 1 eq. of M^{n+} (M^{n+} = Li^+, Na^+, K^+, Cs^+, Mg^{2+}, Ca^{2+}, Sr^{2+}, Ba^{2+}) as their chloride or perchlorate salt to a solution of HNBO or **L1-L3**. All samples were measured 2h after the addition of the metal ion, then 1 eq. of Mg^{2+} was added to each solution to perform ion competition studies. All samples were measured again 2h after the addition of Mg^{2+}. While in the case of **L1** the presence of any A or AE in solution did not alter the emission enhancement obtained upon the addition of Mg^{2+}, for **L2** all A and AE seemed to partially compete with Mg^{2+} (Figure S4; for **L3** see Figure 4a, main text). UV-Vis and fluorescence emission spectra of **L1** + 1 eq. Mg^{2+}, **L2** + 1 eq. Mg^{2+} and **L3** + 1 eq. Mg^{2+} are depicted in Figures S2 and S3 and Figure 3 (main text), respectively.

[1] J. Mohanty, H. Pal, A. V. Sapre, *Bull. Chem. Soc. Jpn.* **1999**, *72*, 2193–2202.

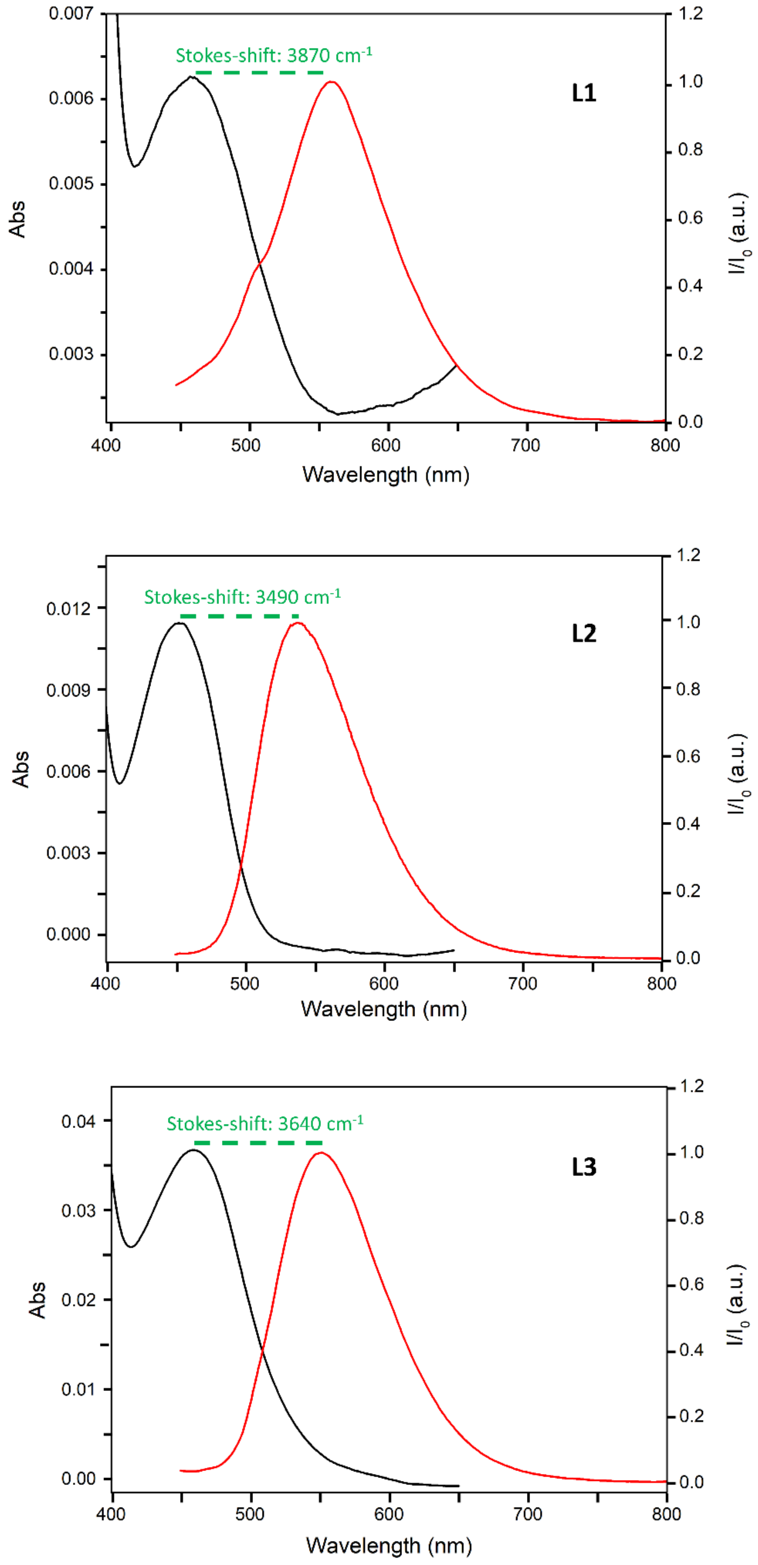

Figure S1. UV-Vis absorption and normalized emission spectra of **L1**, **L2** and **L3** ($1.2 \cdot 10^{-5}$ mol dm^{-3}) in DMSO + 1.5% H_2O, $I = 1.2 \cdot 10^{-3}$ mol dm^{-3} NMe_4Cl. λ_{ex} = 440 nm.

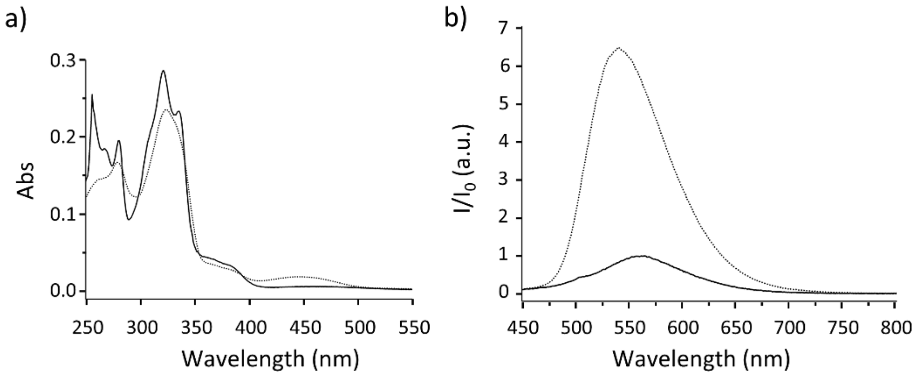

Figure S2. UV-Vis absorption (a) and emission (b) spectra of **L1** ($1.2{\cdot}10^{-5}$ mol dm^{-3}) in DMSO + 1.5% H_2O, I= $1.2{\cdot}10^{-3}$ mol dm^{-3} NMe_4Cl before (solid line) and after (dotted line) the addition of 1 eq. Mg^{2+}. λ_{ex} = 440 nm.

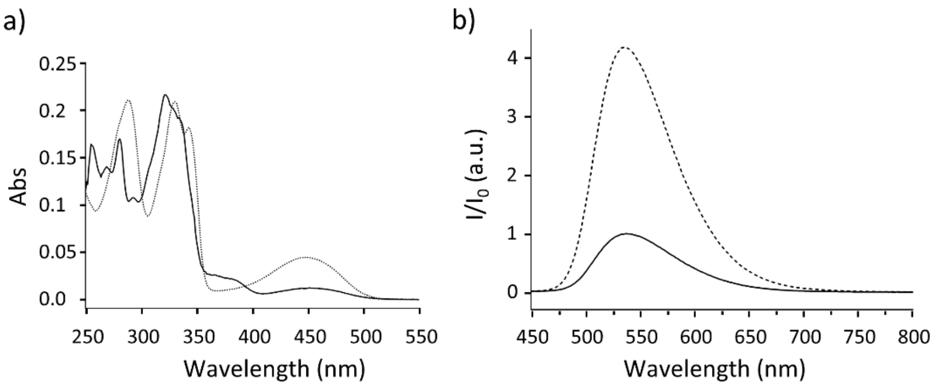

Figure S3. UV-Vis absorption (a) and emission (b) spectra of **L2** ($1.2{\cdot}10^{-5}$ mol dm^{-3}) in DMSO + 1.5% H_2O, I= $1.2{\cdot}10^{-3}$ mol dm^{-3} NMe_4Cl before (solid line) and after (dotted line) the addition of 1 eq. Mg^{2+}. λ_{ex} = 440 nm.

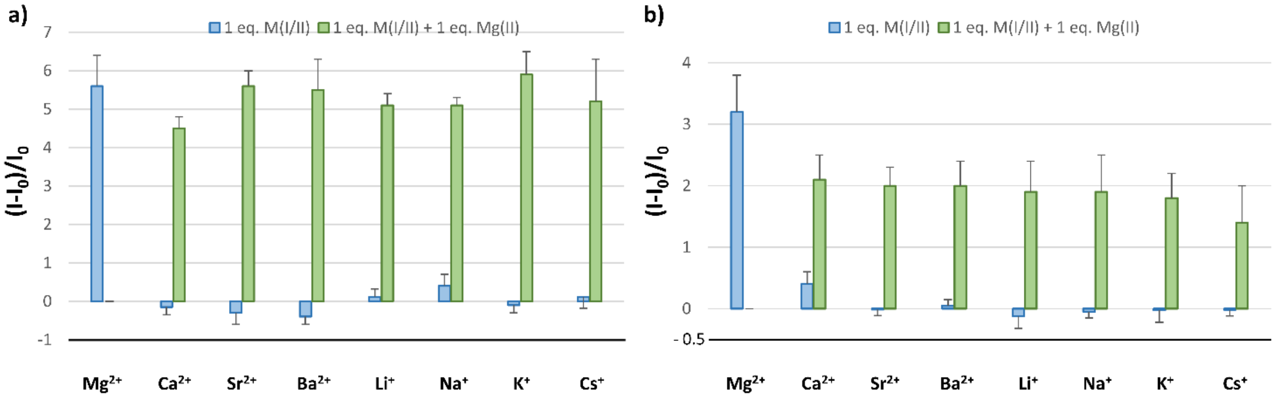

Figure S4. Maximum emission intensity of a) **L1** and b) **L2** upon addition of 1 eq. of A or AE metal ions (blue bars) and further addition of 1 eq. of Mg^{2+} (green bars). **[L]** = $1.2{\cdot}10^{-5}$ mol dm^{-3} in DMSO + 1.5% H_2O, I= $1.2{\cdot}10^{-3}$ mol dm^{-3} NMe_4Cl. λ_{ex} = 440 nm, λ_{em} = 537 nm. Number of replicas: 3.

Preliminary screenings with transition metal ions were carried out by adding 1 equiv. of M^{2+} ($M^{2+} = Zn^{2+}$, Cd^{2+}, Pb^{2+}) as their chloride or perchlorate salt to a solution of **L3**. All samples were measured 2h after the addition of the metal ion, then 1 eq. of Mg^{2+} was added to each solution to perform ion competition studies. All samples were measured again 2h after the addition of Mg^{2+} (Figure S5).

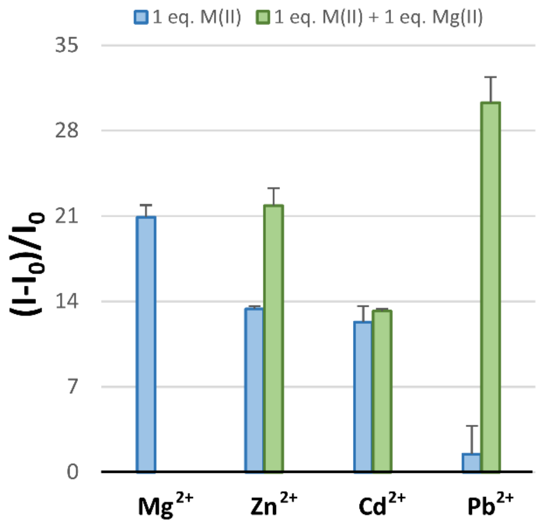

Figure S5. Maximum emission intensity of **L3** upon addition of 1 eq. of transition metal ions (blue bars) and further addition of 1 eq. of Mg^{2+} (green bars). λ_{ex} = 440 nm, λ_{em} = 537 nm (Mg^{2+}), 561 nm (Zn^{2+}), 560 nm (Cd^{2+}), 537 nm (Pb^{2+}). [**L3**] = $1.2 \cdot 10^{-5}$ mol dm^{-3} in DMSO + 1.5% H_2O, $I = 1.2 \cdot 10^{-3}$ mol dm^{-3} NMe_4Cl. Number of replicas: 3.

An additional competition experiment was performed by adding 1 eq. of Mg^{2+} to an equimolar mixture of **L3** and all A and AE metal ions: spectra were acquired before and after the addition of Mg^{2+}, waiting 2h before the acquisition (Figure 4b, main text).

UV-Vis absorption and emission titrations of **L3** with Mg^{2+} were performed by adding to a solution of the ligand (10 cm^3) increasing volumes of a solution of Mg^{2+} (0.1 eq. at a time up to 5 eq.) and keeping the solution mixed for 15 min before starting the acquisition of each spectrum (Figure 3, main text).

Real samples analysis were performed by adding three different brands of commercial water samples and two tap water samples coming from the Italian cities of Urbino and Fano in a quantity corresponding to 1.5% (37.5 μdm^3) to a DMSO solution of **L3** (2.5 cm^3). The same amount of distilled water was added to a DMSO solution of **L3** as a comparison. Florescence emission spectra were acquired 2h after the addition of the sample. The three commercial waters contain different amounts of Mg^{2+}, as from their labels (samples 1 and 2: 4.2, 21.9 ppm, respectively; sample 3: very low, non-reported). The Mg^{2+} content of the two tap water samples was obtained by ion chromatography (see values reported in Figure 5, main text).

To investigate more in depth the mechanism that switches ON the fluorescence of **L3**, the effect of pH has been evaluated, by adding 0.01 mol dm^{-3} HCl/TMAOH to a solution of **L3** in DMSO + 1.5% H_2O (*I*= 1.2·10^{-3} mol dm^{-3} NMe_4Cl) (Figure S6).

At acidic pH the ligand is very low emissive, with the band of the *enol* form (band with λ_{max} at 442 nm) being much higher than that of the *keto* form (band with λ_{max} at 582 nm). It can be hypothesized that the ESIPT process is prevented and the two aromatic systems are not co-planar, with the fluorescence emission of the *enol* form quenched due to TICT formation, since the PET effect, due to the protonated amine functions in this pH field, can be ruled out.

At neutral pH the emission is still low. As observed in the UV-Vis absorption spectrum, a small amount of naphtholate (deprotonated HNBO) form is present (band with λ_{max} at 450 nm) together with the naphthol form (*enol* form: band with λ_{max} at 428 nm; *keto* form is only visible as a tail above 600 nm -; deprotonated form: band with λ_{max} at 545 nm). The deprotonated form is probably responsible for the observed, even if low, fluorescence emission (*vide infra*). A TICT quenching could again be invoked, while a PET mechanism from the free amine function might be probably excluded, since an electron transfer from an amine lone pair towards an anionic emissive excited fluorophore is disfavored.

When **1 eq. of Mg^{2+} is added** to the neutral solution of **L3** an increase of the band with λ_{max} at 450 nm along with a radical modification of the whole UV-Vis absorption spectrum is observed, suggesting the deprotonation of the naphthol unit and the coordination of the metal ion to the HNBO moiety affecting the chromophore transition states. This is accompanied by the switch ON of the fluorescence, with a band having a blue-shifted maximum compared to that at basic pH (λ_{max}: 539 *vs* 568 nm, *vide infra*). The coordination of the metal ion prevents the TICT quenching, forcing the two aromatic systems to be co-planar and switching ON the fluorescence. Moreover, a possible PET effect could be prevented by the coordination of the metal ion, increasing the emission more than that of the deprotonated form at basic pH.

At basic pH, the deprotonated form appears in solution (band with λ_{max} at 465 nm), which is accompanied by the increase of an emission band with λ_{max} at 568 nm; indeed, the deprotonation of the naphthol OH group allows for the delocalization of the negative charge on the whole HNBO system, increasing the electron density on the aromatic rings (that disfavors the PET effect, *vide supra*) and a conjugation-induced co-planarity (that prevents TICT quenching).

Contrarily to the CHEF effect observed at neutral pH, the addition of Mg^{2+} in the opposite acidic and basic pH fields does not alter the fluorescence emission of the ligand, suggesting the lack of Mg^{2+}-coordination in such conditions.

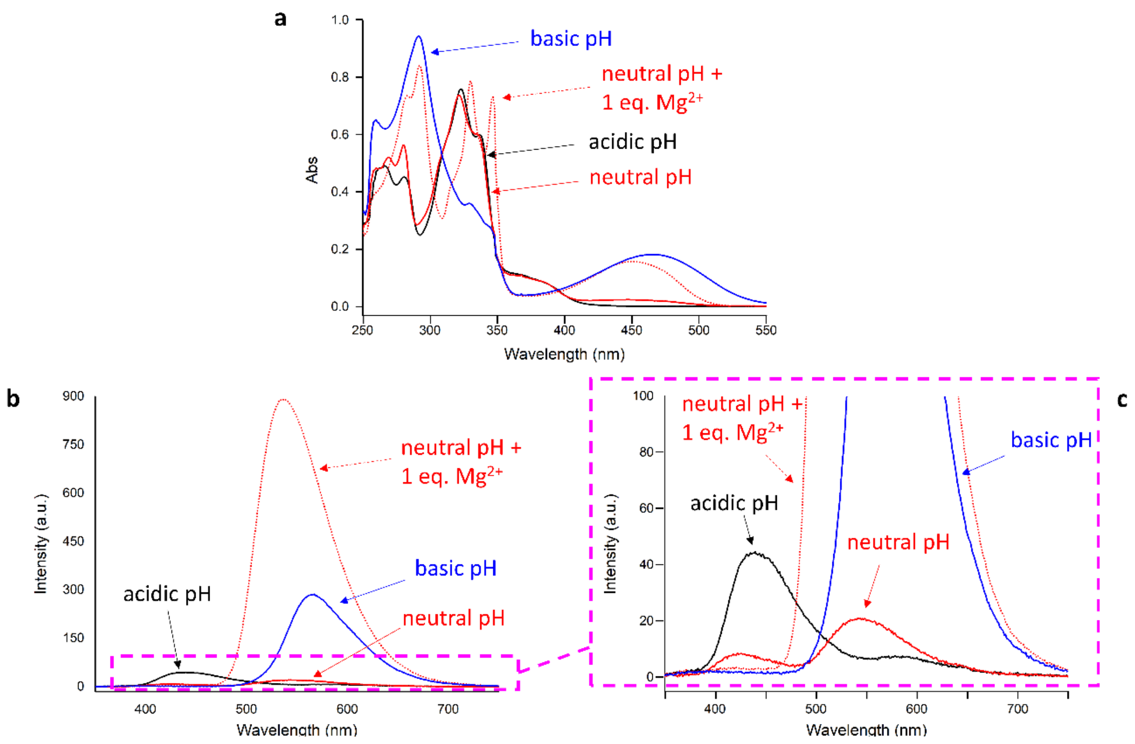

Figure S6. UV-Vis absorption (a) and emission (b; c: zoom) spectra of **L3** ($1.2\cdot10^{-5}$ mol dm^{-3}) in DMSO + 1.5% H_2O, *I*= $1.2\cdot10^{-3}$ mol dm^{-3} NMe_4Cl in acidic (0.01 mol dm^{-3} HCl, black line), neutral (red line) and basic (0.01 NaOH mol dm^{-3}, blue line) pH fields. Addition of 1 eq. Mg^{2+} at neutral pH: red dotted line. λ_{ex} = 322 nm (acidic and neutral pH), 330 nm (basic pH and neutral pH + 1 eq. Mg^{2+}).

NMR spectroscopy

^{1}H and ^{13}C NMR spectra were recorded on a Bruker Avance 400 instrument, operating at 400.13 and 100.61 MHz, respectively, and equipped with a variable temperature controller. The temperature of the NMR probe was calibrated using 1,2-ethanediol as a calibration sample. Chemical shifts (δ scale) are presented in ppm and referenced by residual solvent peak. Coupling constants (*J* values) are given in hertz (Hz). ^{1}H-^{1}H and ^{1}H-^{13}C correlation experiments were performed to assign the signals. All measurements were performed in DMSO-d_6.

^{1}H NMR spectra of **L3** with Mg^{2+} and Ca^{2+} ions were acquired in DMSO-d_6 after the addition of 2 eq. of Bu_4NOH to a $7.5\cdot10^{-3}$ M solution of the ligand. Mg^{2+} and Ca^{2+} ions were then added 0.1 eq. at a time as perchlorate salts dissolved in DMSO-d_6 by direct addition in the NMR tube, that was kept for 10 min at a temperature of 298.1 K before starting the acquisition of each spectrum.

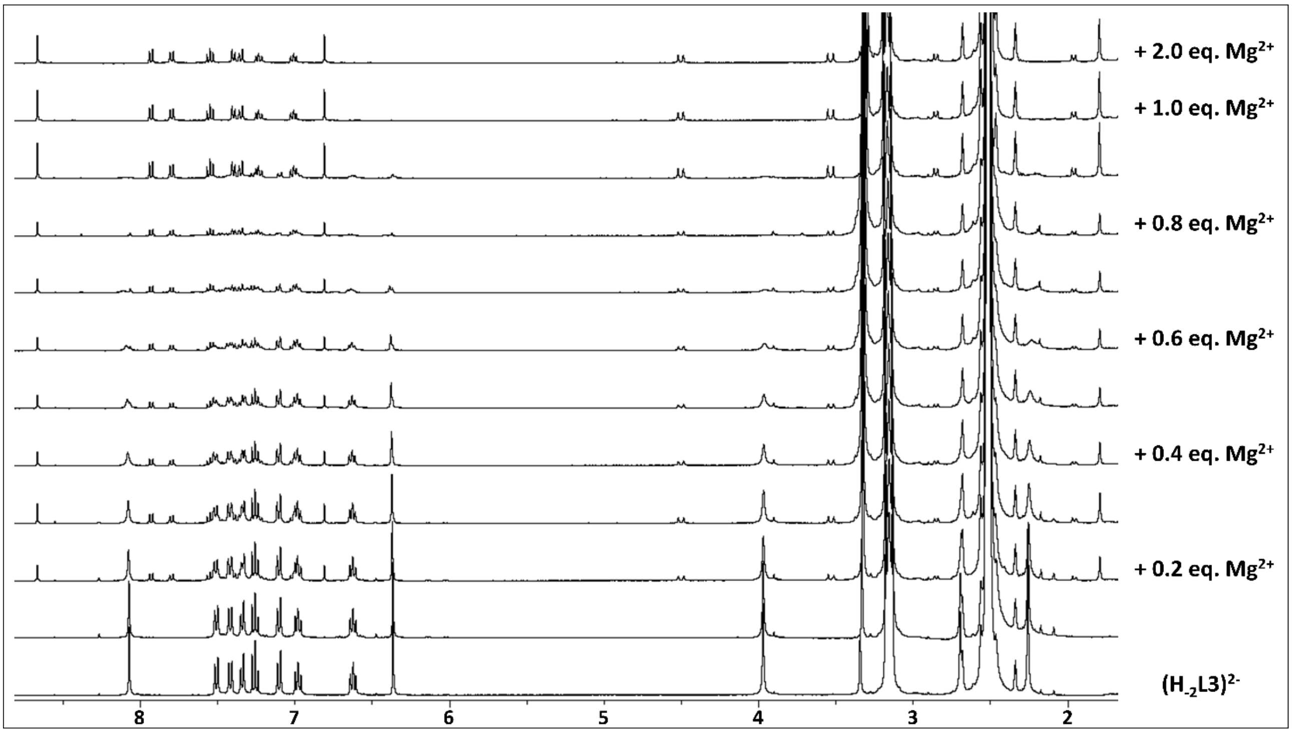

Figure S7. ^{1}H NMR spectra of **L3** registered in DMSO-d_6 in the presence of two eq. of TBAOH upon gradual addition of Mg^{2+}. [**L3**] = $7.5 \cdot 10^{-3}$ mol dm^{-3}.

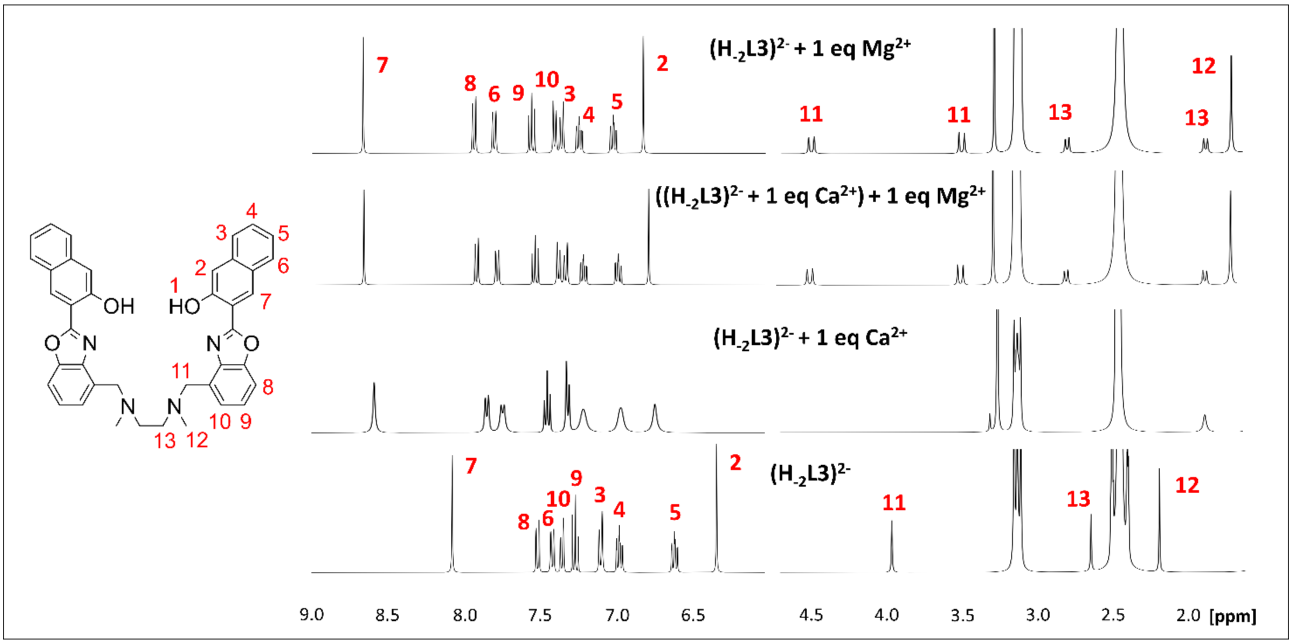

Figure S8. Stacked ^{1}H NMR spectra of $(H_{-2}$**L3**$)^{2-}$, $(H_{-2}$**L3**$)^{2-}$ + 1 eq Ca^{2+}, $(H_{-2}$**L3**$)^{2-}$ + 1 eq Ca^{2+} + 1 eq Mg^{2+}, $(H_{-2}$**L3**$)^{2-}$ + 1 eq Mg^{2+} registered in DMSO-d_6 in the presence of two eq. of TBAOH. [**L3**] = $7.5 \cdot 10^{-3}$ mol dm^{-3}. **L3** atom labelling used in NMR assignments is reported.

Determination of LOD, LOQ and LOL

LOD and LOQ were determined using the linear regression method, that consist in calculating a calibration curve measuring the fluorescence response Y of a series of standard solutions of the analyte of concentration X. This method assumes that the instrument response Y is linearly related to the standard concentration X for a limited range of concentration, and it can be expressed using the equation of the line Y=A+BX as a mathematic model. The two parameters A and B, that are the intercept and the slope of the line, respectively, were calculated with the minimum square method together with their respective standard deviations S_A and S_B. In our case the response is the ratio between the emission intensity of each sample and the emission intensity of the blank at 537 nm by exciting at 440 nm (Figure S5).

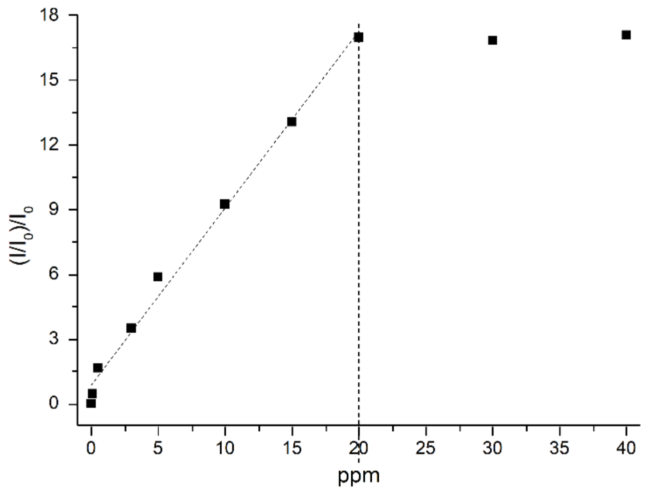

Figure S9. Trend of the normalized emission intensity at 537 nm (λ_{ex}=440 nm) of samples doped with Mg^{2+}.

Membranes preparation

0.5 mg of ligands **L1-L3** have been dissolved into PVC-based polymeric membranes (100 mg total weight) plasticized with 60-66 wt% of tris-octyl phosphate (TOP). The membranes were additionally doped with a lipophilic cation-exchanger (potassium tetra-p-chlorophenyl borate, TpClPBK) in different amounts, as listed in Table S1. All the membrane components were dissolved in 1 dm^3 of THF; membrane cocktails obtained in this way were drop-casted on a FP or CC solid support to obtain an array of several sensing spots and dried until full THF evaporation.

The membranes were conditioned in 0.01 mol dm^{-3} TRIS (pH 8.6) prior to the following measurements to correct the acidity of the solid support. During the tests the mean luminescence value of 6, randomly determined regions of interest of the same size (2 pixels) has been obtained for the same sensing spot.

Table S1. Compositions of the tested **L1-L3**-based polymeric membranes.

Membrane	Ligand, mg	Plasticizer	TpClPBK, eq.
Mb**L1**.1	**L1**, 0.5 mg	TOP	0.5
Mb**L1**.2		TOP	1
Mb**L2**.1	**L2**, 0.5 mg	TOP	0.5
MbL2.2		TOP	3
Mb**L3**.1	**L3**, 0.5 mg	TOP	0.5
Mb**L3**.2		TOP	2

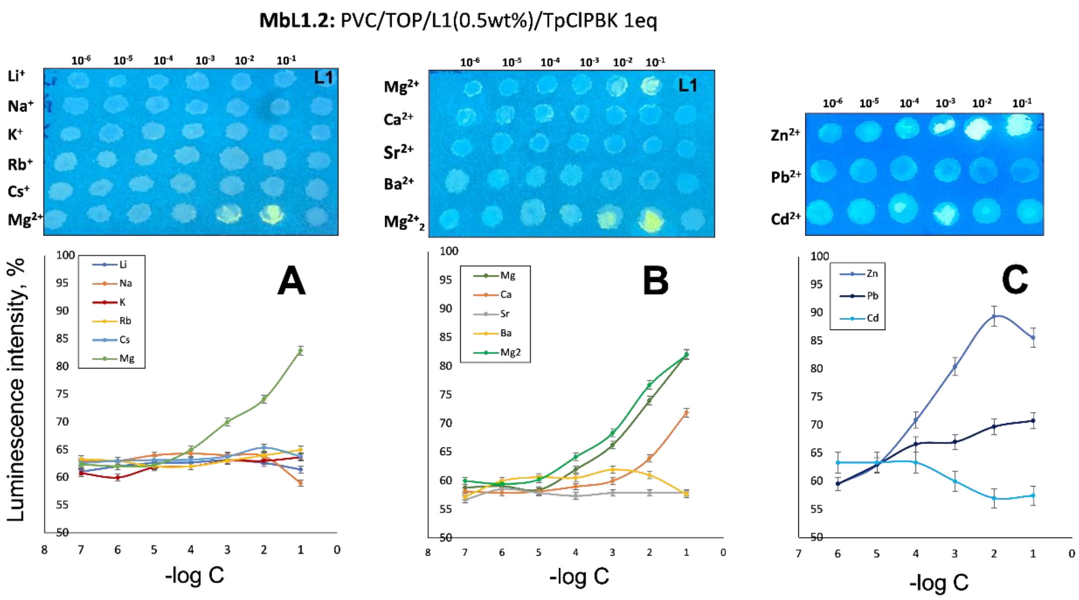

Figure S10. The response of **L1**-based optical sensing spot arrays to a) Alkali , b) Alkaline-Earth and c) Zn^{2+}, Pb^{2+} and Cd^{2+} metal cations (λ_{ex} = 365 nm). Top: photographs of sensing spots deposited on CC support; bottom: calibration curves representing the relative luminescence intensity (in %) of Mb**L1**.2-based optode to growing concentrations (-log C) of tested ions. Number of replicas: n=6.

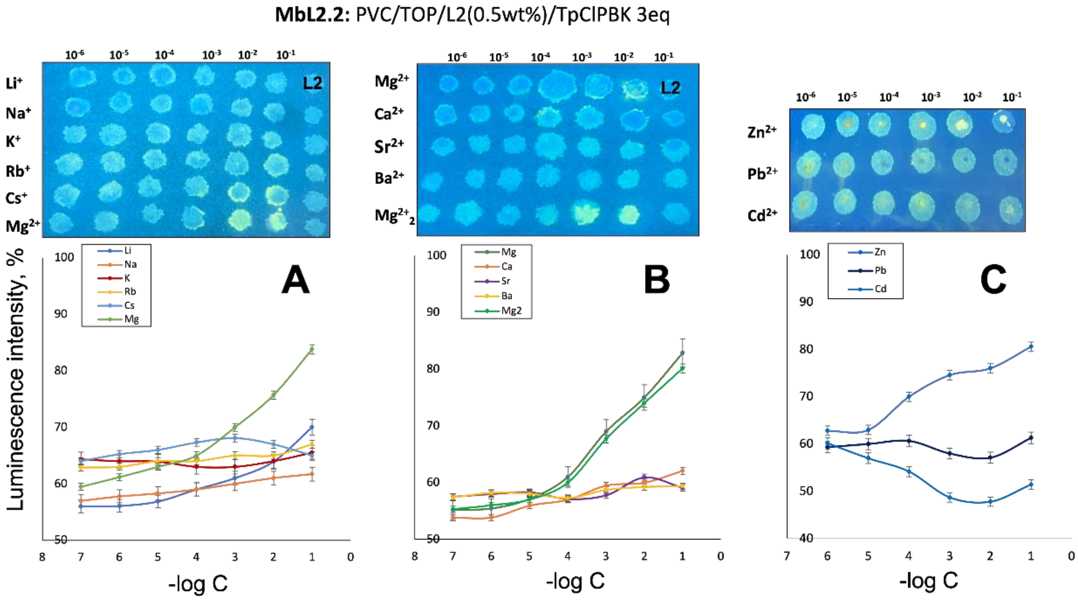

Figure S11. The response of **L2**-based optical sensing spot arrays to a) Alkali, b) Alkaline-Earth and c) Zn^{2+}, Pb^{2+} and Cd^{2+} metal cations, (λ_{ex} = 365 nm). Top: photographs of sensing spots deposited on CC support; bottom: calibration curves representing the relative luminescence intensity (in %) of Mb**L2**.2-based optode to growing concentrations (-log C) of tested ions. Number of replicas: n=6.

Chemometric data treatment

The Principal Component Analysis (PCA) was applied to interpret the optical output of the sensor array based on ligands **L1-L3** employed for the analysis of multi-ion solutions mimicking the composition of natural waters. Data treatment was performed with commercial Unscrambler (v. 9.1, 2004, CAMO PROCESS AS, Norway). Due to the restricted number of analyzed samples the validation was performed by using a leave-one-out cross-validation procedure.

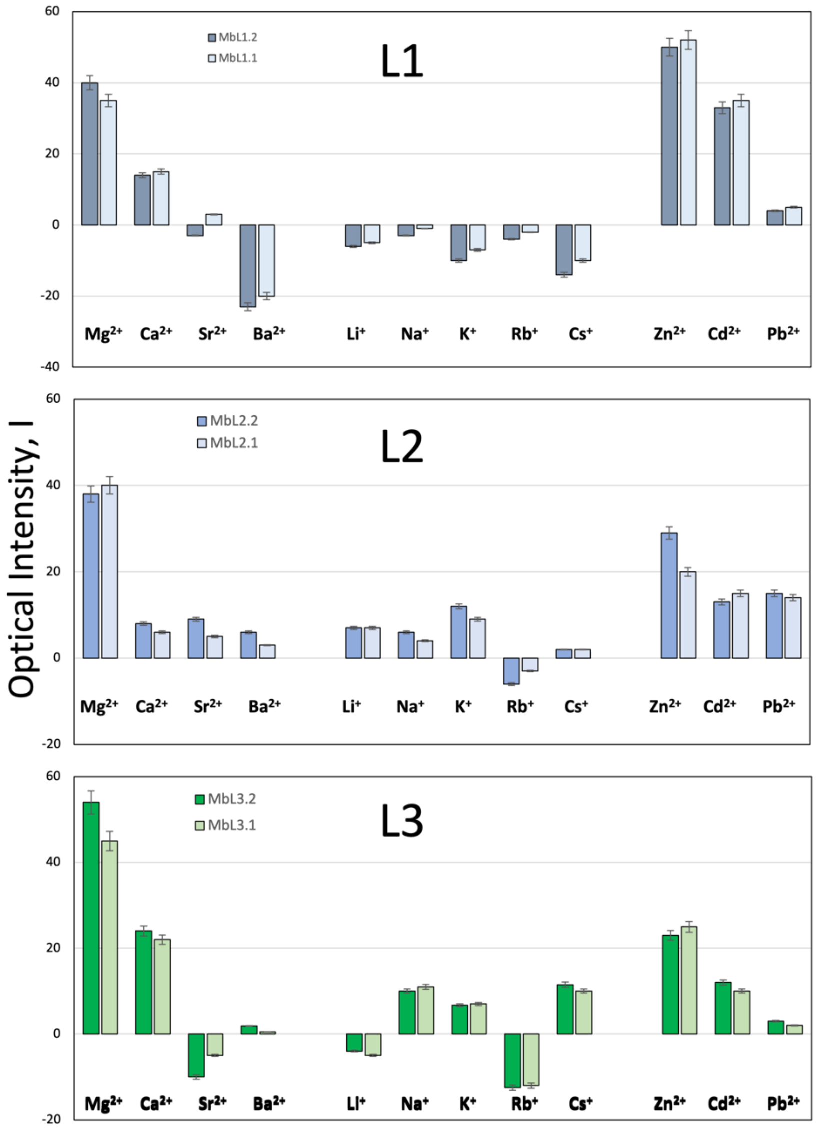

Figure S12. Optical luminescence response of **L1-L3**-based membranes in individual 0.01 mol dm^{-3} aqueous solutions containing Alkali, Alkaline-Earth and Zn^{2+}, Cd^{2+} and Pb^{2+} ions. Luminescence evaluated at 365 nm, number of replicas: n=6.

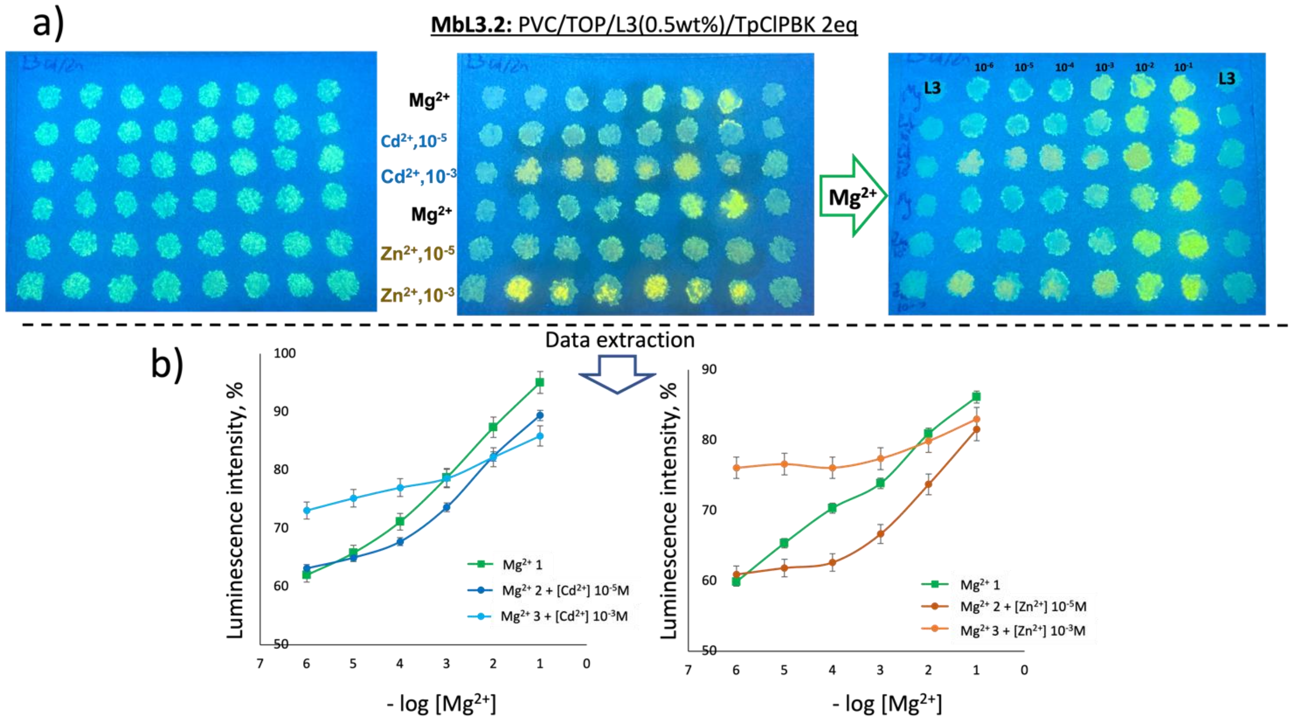

Figure S13. a) Photographs of **L3**-based optodes soaked in 0.01 mol dm^{-3} TRIS pH 8.6 (left), after addition of individual aqueous solutions containing Cd^{2+} and Zn^{2+} ions in concentrations 10^{-5} and 10^{-3} mol dm^{-3} (top and bottom spot rows, respectively) (middle), and consecutive addition of Mg^{2+} ions in concentration range from 10^{-6} to 10^{-1} mol dm^{-3} (right); b) calibration curves representing the relative luminescence intensity (in %) of Mb**L3**.2-based optode to growing concentrations of Mg^{2+}-ions (-log [Mg^{2+}]) in the presence of Cd^{2+} and Zn^{2+} ions. Number of replicas: n=6 (λ_{ex} = 365 nm).

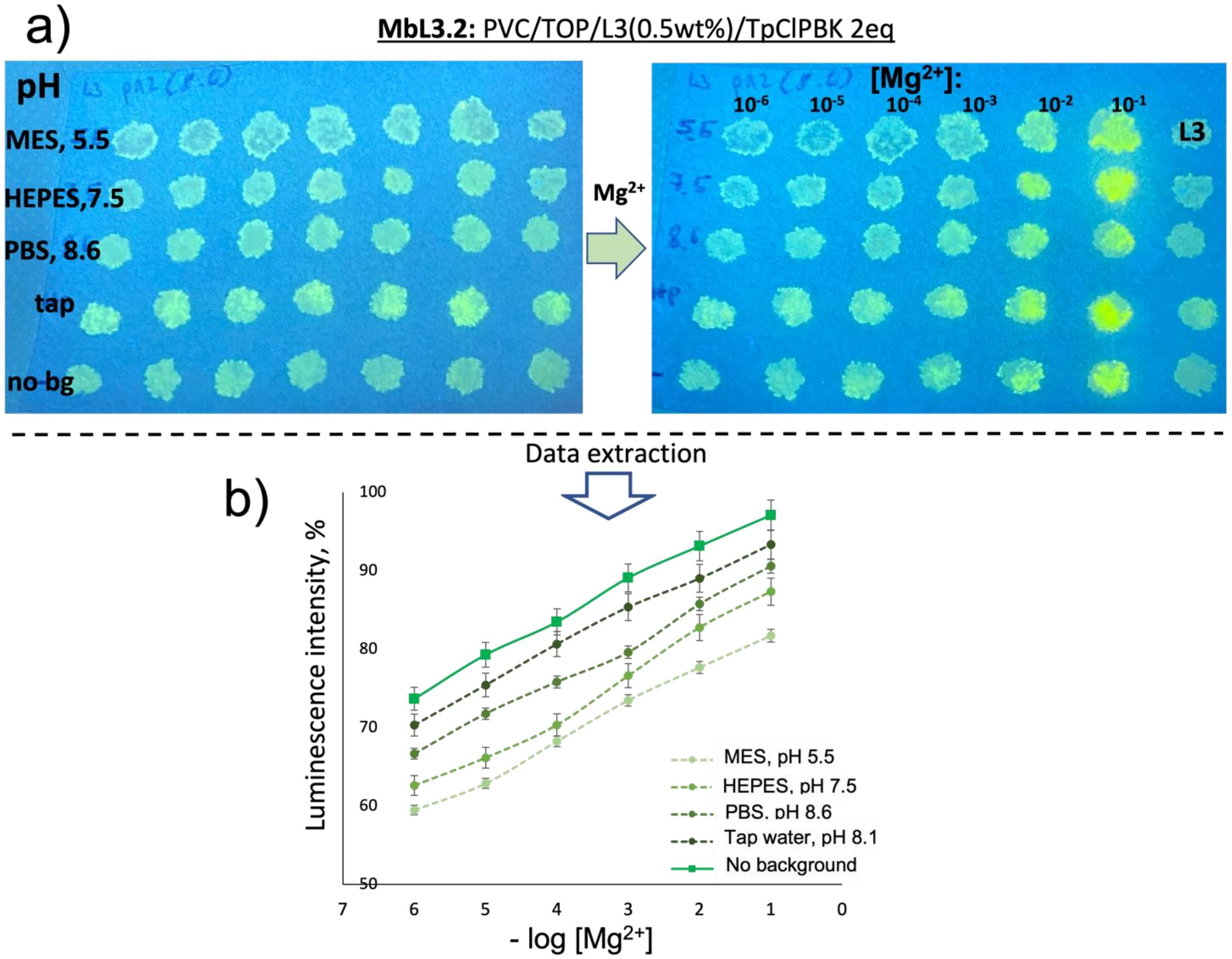

Figure S14. a) Photographs of **L3**-based optodes soaked in 0.01 mol dm^{-3} TRIS pH 8.6, after addition of 0.01 mol dm^{-3} solutions of (from top to bottom): MES pH 5.5, HEPES pH 7.5, PBS pH 8.6, tap water pH 8.1 and without treatment (left) and after addition of Mg^{2+} ions in concentration range from 10^{-6} to 10^{-1} mol dm^{-3} (right); b) calibration curves representing the relative luminescence intensity (in %) of Mb**L3**.2-based optode to growing concentrations of Mg^{2+}-ions (-log [Mg^{2+}]) on different pH backgrounds. Number of replicas: n=6 (λ_{ex} = 365 nm).

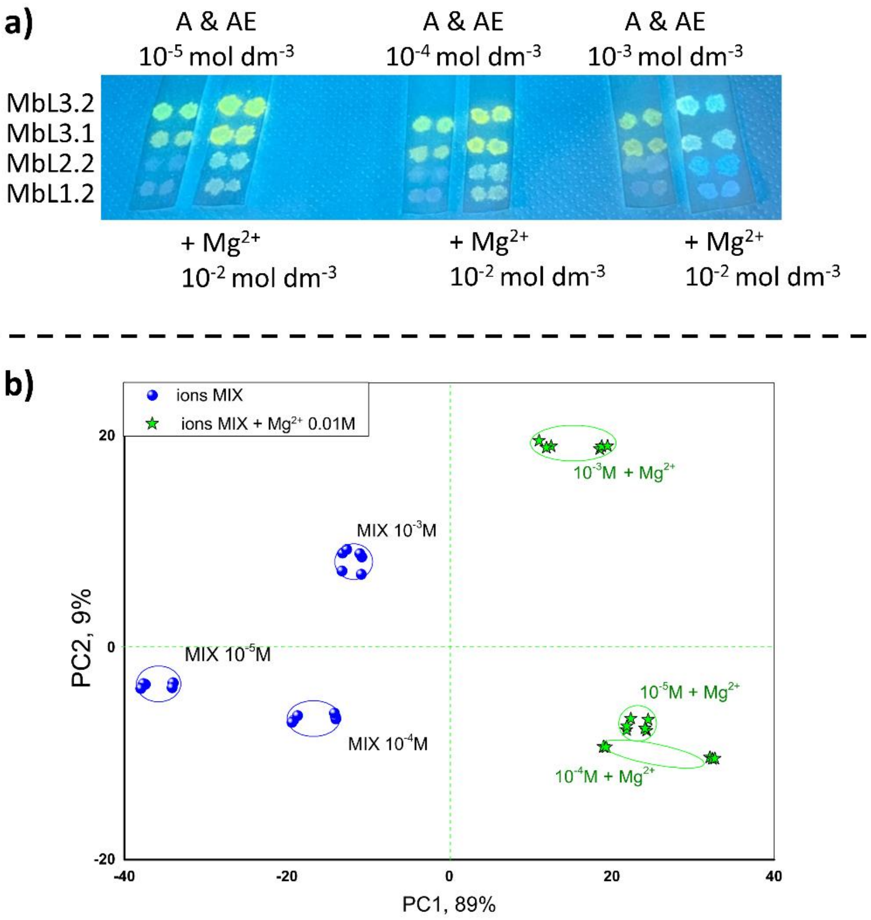

Figure S15. a) Photograph of **L1-L3**-based optodes in multicomponent model solutions mimicking natural waters containing Alkali and Alkaline-Earth in various concentrations (10^{-5}, 10^{-4}, 10^{-3} mol dm^{-3}) without Mg^{2+} ions and the same solutions with the control addition of $1 \cdot 10^{-2}$ mol dm^{-3} Mg^{2+} ions. b) PCA score plot of the **L1-L3**-based sensor array in multicomponent model solutions (λ_{ex} = 365 nm).
